# Lung contusion and cavitation with exudative plural effusion following extracorporeal shock wave lithotripsy in an adult: a case report

**DOI:** 10.1186/1752-1947-4-293

**Published:** 2010-08-31

**Authors:** Nader Nouri-Majalan, Roghayyeh Masoumi, Abolhasan Halvani, Sara Moghaddasi

**Affiliations:** 1 Nephrology Department, Sadoughi Medical University, Yazd, Iran; 2 Pulmonary Department, Sadoughi Medical University, Yazd, Iran

## Abstract

**Introduction:**

Among the complications of extracorporeal shock wave lithotripsy are perinephric bleeding and hypertension.

**Case presentation:**

We describe the case of a 31-year-old Asian man with an unusual case of hemoptysis and lung contusion and cavitation with exudative plural effusion due to pulmonary trauma following false positioning of extracorporeal shock wave lithotripsy. Differential diagnoses included pneumonia and pulmonary emboli, but these diagnoses were ruled out by the uniformly negative results of a lung perfusion scan, Doppler ultrasound, and culture of bronchoalveolar lavage and plural effusion, and because our patient showed spontaneous improvement.

**Conclusions:**

False positioning of extracorporeal shock wave lithotripsy can cause lung trauma presenting as pulmonary contusion and cavitation with plural effusion.

## Introduction

Although extracorporeal shock wave lithotripsy (ESWL) is useful for the management of renal calculi, it is associated with several side effects, including subcapsular and perinephric bleeding [1], hypertension [2] and splenic hematoma [3]. ESWL has also been reported to be associated with rare pulmonary complications, including pulmonary contusion [4], pulmonary edema [5] and hemoptysis in a child [6]. Here, we report an unusual case of hemoptysis following ESWL.

## Case presentation

A 31-year-old Asian man with a history of asthma presented with left pleuritic chest pain.

One week earlier, he had suffered from renal colic in the left flank. Ultrasound showed an 11 mm stone in the proximal section of the left ureter. He underwent ESWL, consisting of 4000 lithotripsy shocks at 87 kV, administered with a Delta Dornier lithotripter (Dornier Medical Systems, Marietta, GA). Two days later, our patient passed the stone, accompanied by renal colic. At that time, however, he had no pulmonary symptoms.

Physical examination showed diminished breath sounds in his left lung, accompanied by generalized wheezing. His blood pressure was 120/80 mm/Hg and his respiratory and heart rates were 15 and 80 per minute, respectively. His body temperature was 37.2°C. Blood biochemistry revealed a white blood cell count (WBC) of 10,800 cell/mL, hemoglobin (HG) of 14.3 g/dL, platelets (PLT) of 365,000/mL, erythrocyte sedimentation rate (ESR) of 87 mm/h, and D-dimer of 2 ng/mL. Analysis of his pleural fluid showed WBC of 2000/mL, red blood cell count (RBC) of 600/mL, glucose 79 mg/dL, protein 4.8 g/dL and Lactate dehydrogenase (LDH) of 984 U/L. His pleural fluid LDH/serum LDH ratio was 984/930. Gram staining and cytology of his pleural effusion fluid showed no evidence of microorganisms or malignancy. Analysis of his arterial blood gas showed a pH of 7.37, a pO_2 _of 63.5 mmHg, O_2 _saturation of 91.2%, a pCO_2 _of 43 mmHg and HCO_3 _of 24.6 meq/L.

**Figure 1:**
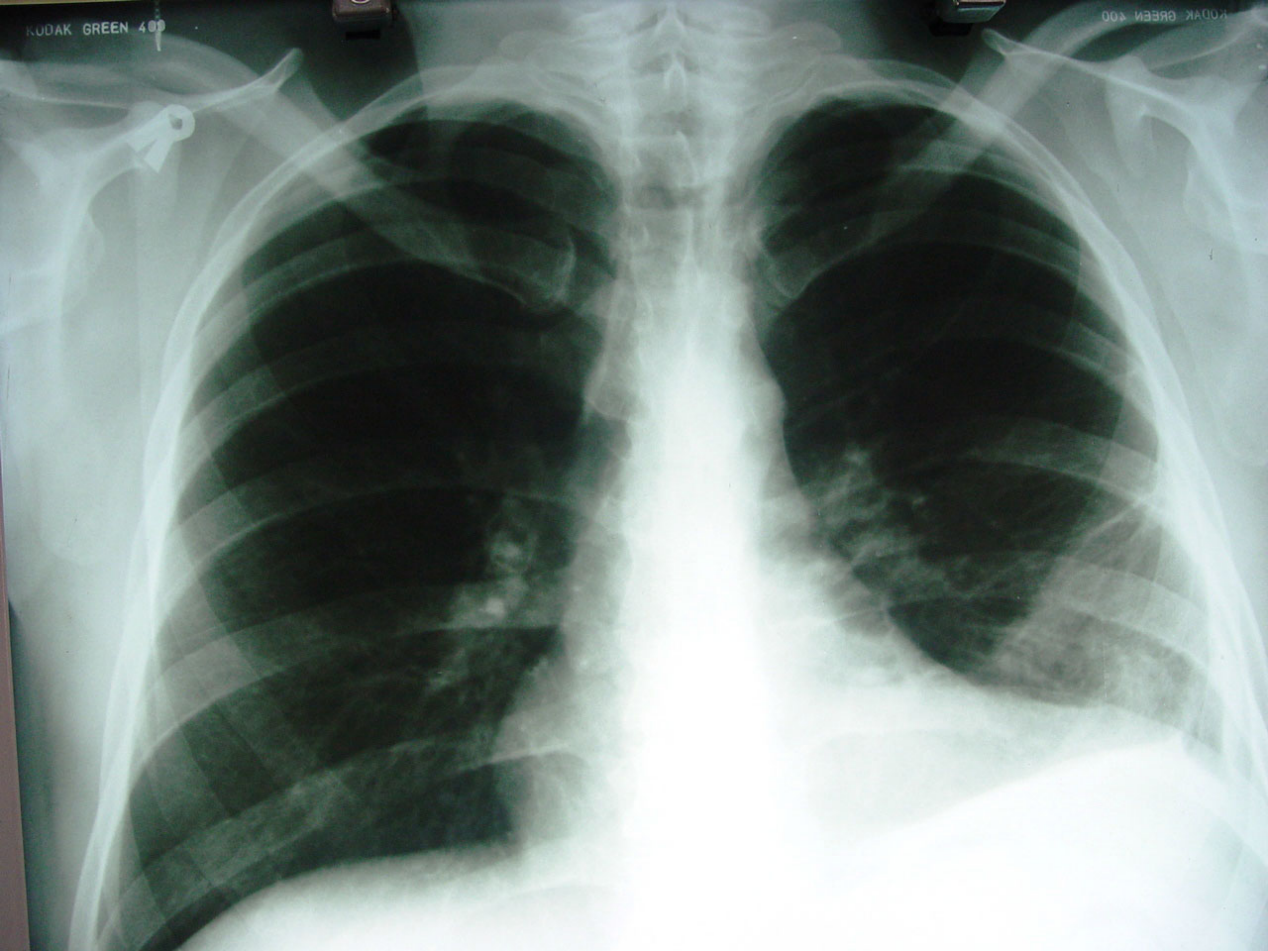
**Chest X-ray showing consolidation in the lower lobe of the left lung**.

**Figure 2:**
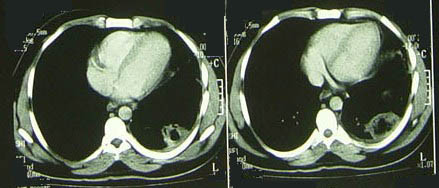
**CT scan showing left lower lobe consolidation with cavitation and moderate pleural effusion**.

Chest X-ray and a chest computed tomography (CT) scan showed consolidation with cavitation in the lower lobe of the left lung and moderate plural effusion on the left side (Figures 1 and 2). A perfusion lung scan revealed decreased perfusion in the subsegment of the left lung, indicating a low probability of pulmonary emboli. Lower extremity venous ultrasound showed no evidence of thrombosis. Culture of bronchoalveolar lavage (BAL) samples showed no evidence of microorganisms.

Our patient was treated with antibiotics and heparin for two days. After obtaining BAL culture and lung scan results, however, treatment was discontinued. He recovered spontaneously after four days.

## Discussion

To the best of our knowledge, this is the first report in an adult of pulmonary contusion and cavitation with exudative plural effusion due to lung trauma following false positioning of ESWL. Differential diagnoses included pneumonia and pulmonary emboli, but these diagnoses were ruled out because the results of lung perfusion scan, Doppler ultrasound, and culture of BAL and plural effusion were all negative, and because our patient showed spontaneous improvement.

Although hemoptysis following ESWL usually starts during or shortly after the procedure [4,6], our patient first showed evidence of hemoptysis one week after ESWL. Chest radiography and CT scan showed lung consolidation with cavitation and pleural effusion; in previous patients, chest X-rays were normal [7] or showed only lung contusion [4]. Two children with lithotripsy-induced pulmonary contusion and hemoptysis have been described [4-7]. Due to their smaller body surface area and the shorter distance between the lung base and the kidney, the likelihood of pulmonary trauma following ESWL may be higher in children than in adults [8].

Pulmonary contusion following ESWL has also been shown experimentally in mice [9]. At the microscopic level, shock waves have been shown to cause trauma in pneumocytes and endothelial cells, resulting in a direct communication between the lumina of vessels and alveolar spaces, ultimately leading to hemoptysis [10].

Life threatening hypoxemia following ESWL has also been reported [11]. Our patient, however, had moderate hypoxemia.

## Conclusions

False positioning of ESWL can cause lung trauma presenting as pulmonary contusion and cavitation with plural effusion.

## Competing interests

The authors declare that they have no competing interests.

## Authors' contributions

NN was primarily responsible for the diagnosis and management of the patient, drafting of the manuscript, literature search, and submission and revision of the manuscript. RM and SM were responsible for drafting of the manuscript and literature search. AH was responsible for the diagnosis and management of the patient. All authors have read and approved the final manuscript.

## Consent

Written informed consent was obtained from the patient for publication of this case report and any accompanying images. A copy of the written consent form is available for review by the Editor-in-Chief of this journal.
